# Ultrarobust subzero healable materials enabled by polyphenol nano-assemblies

**DOI:** 10.1038/s41467-023-36461-9

**Published:** 2023-02-13

**Authors:** Nan Wang, Xin Yang, Xinxing Zhang

**Affiliations:** State Key Laboratory of Polymer Materials Engineering, Polymer Research Institute, Sichuan University, 610065 Chengdu, China

**Keywords:** Composites, Sensors and biosensors, Nanoparticles, Molecular self-assembly

## Abstract

Bio-inspired self-healing materials hold great promise for applications in wearable electronics, artificial muscles and soft robots, etc. However, self-healing at subzero temperatures remains a great challenge because the reconstruction of interactions will experience resistance of the frozen segments. Here, we present an ultrarobust subzero healable glassy polymer by incorporating polyphenol nano-assemblies with a large number of end groups into polymerizable deep eutectic solvent elastomers. The combination of multiple dynamic bonds and rapid secondary relaxations with low activation energy barrier provides a promising method to overcome the limited self-healing ability of glassy polymers, which can rarely be achieved by conventional dynamic cross-linking. The resulted material exhibits remarkably improved adhesion force at low temperature (promotes 30 times), excellent mechanical properties (30.6 MPa) and desired subzero healing efficiencies (85.7% at −20 °C). We further demonstrated that the material also possesses reliable cryogenic strain-sensing and functional-healing ability. This work provides a viable approach to fabricate ultrarobust subzero healable glassy polymers that are applicable for winter sports wearable devices, subzero temperature-suitable robots and artificial muscles.

## Abstract (web summary)

Self-healing materials hold great promise for applications in wearable electronics, artificial muscles and soft robots but selfhealing at subzero temperatures remains a great challenge. Here, the authors present a robust subzero healable glassy polymer by incorporating polyphenol nano-assemblies with a large number of end groups into polymerizable deep eutectic solvent elastomers.

## Introduction

Animal skin is an intriguing biological tissue that has long captivated the scientific community due to its high toughness, self-healing properties when damaged, and ability to transmit information^1–3^. Inspired by this, many kinds of self-healing materials have been designed through the introduction of dynamic bonds or preembedded healing reagents, which hold broad potential applications in wearable electronics, human–machine interfaces, robotics, etc.^4–7^. Wearable electronics are prone to cracks and functional failures under repeated stretching and torsion, which is more likely to occur under subzero conditions^8–12^. However, processes such as the movement and conformational transition of the chain segments become challenging at low temperatures, resulting in the huge limitation of self-healing ability^13^.

Due to the resisted dynamic characteristics of polymers at subzero temperatures, external input of energy, such as thermal stimulus, high pressure, or the assistance of solvents are usually required to heal, which greatly limits their practical applications^14–16^. To realize reversible and autonomous self-healing properties at low temperatures, especially freezing conditions, fast dissociation–reconstruction reactions with low activation energy barriers are generally required. This process is usually together with a low glass-transition temperature (*T*_g_) to enable polymer chains with mobility. Therefore, self-healing ability below zero is generally reported in hydrogels and elastomers^17,18^. For example, there is some research about polymers that can be self-healed below −20 °C through dense hydrogen bonding networks and the ultra-flexible molecular chains design^19^. But their *T*_g_ is much lower than the temperature where they are capable of self-healing, resulting in limited mechanical strength. For glassy polymers with high mechanical strength, the ability to self-heal at subzero temperatures becomes even more out of reach^20,21^. Therefore, it is still a great challenge to develop a subzero self-healing glassy polymer with high mechanical strength.

Recently, due to the advantages of simple, solvent-free preparation and high ionic conductivity, the newly emerging type of polymerizable deep eutectic solvent (PDES) has attracted the attention of functional materials^22,23^. PDES are usually obtained by the complexion of hydrogen bond acceptors, mainly quaternary ammonium salts, with hydrogen bond donors. Abundant hydrogen bonding sites and ionic conductivity endow it with potential applications in self-healing or sensing materials. However, this kind of polymer is still not satisfactory for the fabrication of subzero healable materials, because of the frozen state of molecular chains with limited thermal motion energy under low temperature^24^.

Herein, we propose an ultra-robust subzero self-healing material enabled by polyphenol nano-assemblies. The key to this design is that a large number of end groups and branch units are constructed on the nano-assemblies and PDES molecular chains, respectively, allowing secondary relaxations to happen with low activation energy barriers. In addition, reversible metal coordination and hydrogen bonds built between these units are expected to provide sufficient interfacial interactions. The complementarity and dynamics are achieved by their orderly combination^25^, which endows the materials with excellent strong mechanical properties and self-healing properties at subzero temperatures. Furthermore, we also explore the strain-sensing ability and functional healing performance of the material below 0 °C. This work will provide valuable guidance for the development of electronic skins (e-skins) with high-performance stability over a wide temperature range, which can be used in wearable monitoring devices for winter sports, subzero temperature-suitable robots, and artificial muscles.

## Results

### Coordination-regulated assembly of polyphenol nanospheres

**Fig. 1:**
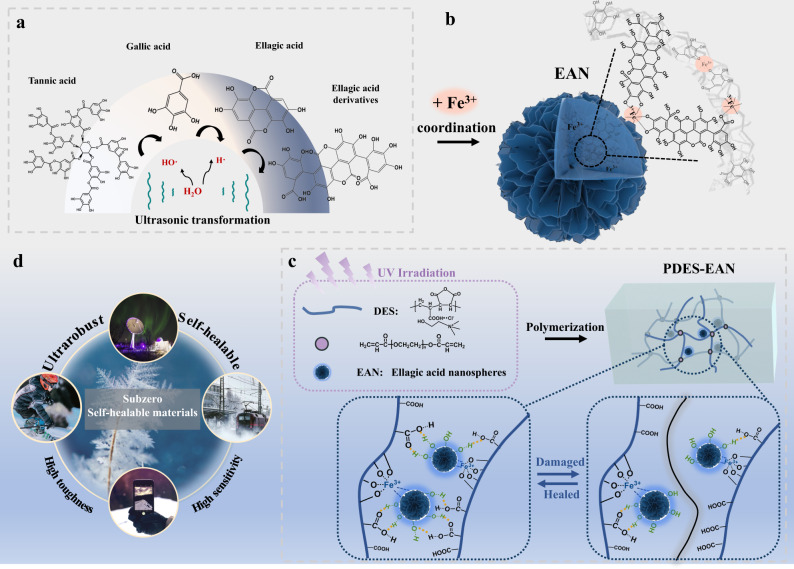
Materials design of the subzero healable glassy polymer. **a** Schematic illustration of the mechanism for the ultrasonic transformation of TA to EA. **b** Schematic illustration of EAN structure. **c** Schematic illustration of the photopolymerization of PDES and the synergistic interaction consisting of metal–ligand coordination and hydrogen bonding of EAN and PDES matrix, which enable the self-healing ability of PDES–EAN. **d** Potential applications of the material including wearable devices, electronics, structural materials, and polar exploration devices in subzero conditions.

To overcome the challenge of fabricating glassy polymers with self-healing ability at subzero temperatures, a nano-scale assembly possessing multiple and cooperative dynamic bonds is proposed as shown in Fig. 1. Tannic acid (TA) is a natural metabolite derived from plants such as grapes, green tea, coffee, and nuts, which has multiple gallic acid units attached to the central sugar core. Under ultrasonication, TA undergoes molecular reorganization through hydrolyzed into gallic acid (GA) first and then forms sheet-like ellagic acid (EA) structures through C–C coupling of phenolic moieties^26^. This process achieves the transformation of TA molecules from large sterically hindered three-dimensional structures to small planar structures, accompanied by abundant phenolic hydroxyl groups at the edges. Fe^3+^ is then introduced to penetrate between EA molecules, and induced the complexation of EA molecules into aggregated nano-spheres (EAN) through metal–hydroxyl interactions^27–30^. The obtained nano-assemblies possess a large number of highly active end groups, making it possible to construct a dynamic network of hydrogen bonds and metal coordination bonds with low activation energy.

**Fig. 2:**
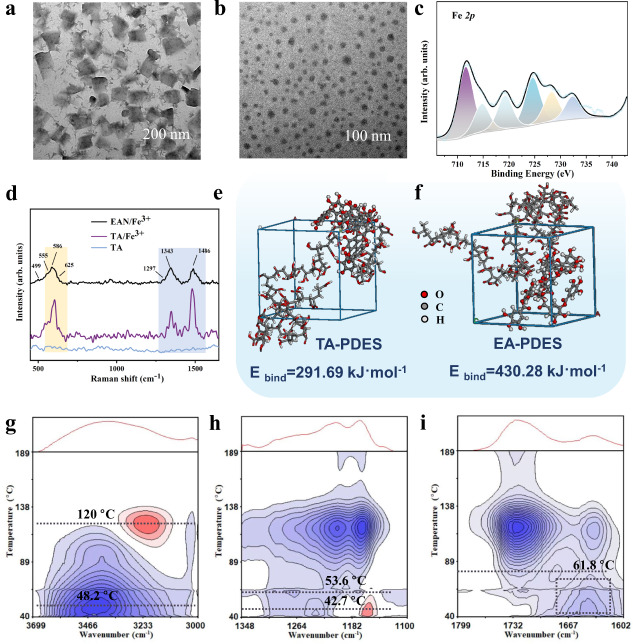
Characterization of the EAN structure and dynamic multiple hydrogen-bonding networks. **a** The transmission electron microscopy (TEM) image of brick-like crystals formed by EA stacking. **b** The TEM image of EAN with coordination regulation of Fe^3+^. **c** The Fe 2*p* segment in the XPS spectra of EAN. **d** Laser confocal Raman spectra of TA and EAN. **e**, **f** Binding energy simulation models of TA–PDES composites and EA–PDES composites after molecular dynamic simulation. **g–i** PCMW2D spectra of PDES–EAN.

As shown in Fig. 2a, b, EANs show regular spherical shapes constrained by the coordination of Fe^3+^, which are significantly different from the submicron sheets and brick-like crystals of EA-only assemblies. The good dispersion of EANs in the polymer system with small and uniform particle sizes of about 60–140 nm (Supplementary Fig. 1) provides a basis for the construction of a physical cross-linking network. Compared with the sheet and brick-like or sterically hindered three-dimensional structures, spherical nano-assemblies possess better mobility in the polymer matrix and larger specific surface area, thus providing more abundant hydrogen bonding sites. X-ray spectroscopy (XPS) peaks in 710–735 eV were identified as the region of Fe 2*p*^31,32^, shown in Fig. 2c. The O 1*s* region located in 530–536 eV (Supplementary Fig. 2, Supplementary Data 1) is fitted to two peaks, which are assigned to the –C=O and –C–O– groups. In addition, according to the laser confocal Raman microspectroscopy, the binds at 1297, 1343, 1486 cm^−1^ belong to the ring vibrations of EA, while the bands at 499, 555, 586, 625 cm^−1^ are attributed to the interactions between Fe^3+^ and the phenolic oxygens of the catechol^33,34^ (Fig. 2d, Supplementary Data 2). The peak location is consistent with TA/Fe^3+^. It indicates the formation of metal–ligand coordination bonds inside EAN, further demonstrating the regulation of Fe^3+^ in the assembly process.

With the frozen network of glassy polymers at low temperatures, it is of benefit to construct abundant end groups and branch units with high mobility for cryogenic self-healing. Copolymerization is an attractive approach to prepare PDES by incorporating soft acrylic acid (AA) and hard maleic anhydride (MAH) segments as hydrogen bond donors^35,36^. Choline chloride (ChCl) is chosen as a hydrogen bond acceptor to provide ionic conductivity. Importantly, the regular and comb-like branch units (–COOH) from the backbone of PDES are able to form dense hydrogen bonds with the abundant end groups (–OH) of EAN. The residual Fe^3+^ on the surface of EAN is also able to form coordination with –COOH groups. The high-density interactions of these end groups and branched units possess great potential for improving the secondary transformation activity of molecular groups. In a word, the dynamic cross-linking network is constructed with low-activation energy to enable cryogenic self-healing by constructing rich end groups (–OH) and branch units (–COOH) on the nanospheres and polymer molecular chains, respectively^37^.

### Interfacial dynamic bonds network

In order to further understand the interaction mechanism of hydrogen bonds and obtain the optimized conformation between PDES molecular chain and TA molecule or EA nanosheet with energy minimization (Fig. 2e, f), isothermal-isovolumic molecular dynamics simulations of the composite model were performed^38,39^. The binding energy can be calculated according to the following equation:

1 $${E}_{{{\rm {bind}}}}={E}_{{{\rm {polymer}}}}+{E}_{{{\rm {filler}}}}-{E}_{{{\rm {total}}}}$$

in which *E*_polymer_ and *E*_filler_ present the corresponding energy of the PDES and EA in the equilibrium conformation; *E*_bind_ presents the binding energy between EA and PDES matrix. The results show that the binding energy between PDES and EA reaches up to 430.28 kJ mol^−1^, whereas the binding energy of PDES and TA is 291.68 kJ mol^−1^. The difference in the binding energy indicates that under the condition of the same number of GA units, the lamellar structure after the ultrasonic transformation has greater interfacial interaction with the matrix than the original large sterically hindered branched structure, thus leading to the remarkable self-healing ability and mechanical strength.

Furthermore, temperature-dependent Fourier transform infrared (FTIR) spectroscopy was performed to characterize the multiple hydrogen bonding of PDES–EAN nanocomposites (Supplementary Data 3). The peak centered at 3436 cm^−1^ is belong to the stretching vibration band of –OH (Supplementary Figs. 3–5). It is worth noting that this peak is very broad, indicating the coexistence of multiple hydrogen bonds in PDES–EAN molecules. As the temperature increases, the intensity decreases obviously, showing the dissociation of a large number of hydrogen bonds upon heating. However, the intensity of higher wavenumber belongs to the free –OH group band keeps decreasing, which indicates that no free –OH groups are generated during heating. At the same time, the intensity around 3240 cm^−1^ increases gradually, which suggests that –OH groups tend to form metal coordination with Fe^3+^ in the high-temperature region. The peaks located at 1645 and 1790 cm^−1^ can be assigned to the –C=O groups of EAN and PDES, respectively, and the intensity decreases significantly with increasing temperature. Based on the results of temperature-dependent FTIR, the perturbation-correlation moving-window 2D (PCMW 2D) spectra of PDES–EAN were obtained to figure out the specific temperature of multiple hydrogens dissociation^40^. Six correlation cross peaks assigned to –OH, –C=O, and –C–O–C moieties are visible in 1100–3700 cm^−1^, shown in Fig. 2g–i. The results show that hydrogen bonds in this system are highly active and its dissociation can be achieved over a wide temperature range, which lays a foundation for the self-healing ability of PDES–EAN nanocomposites.

### Activity of dynamic bonds at low temperature

**Fig. 3:**
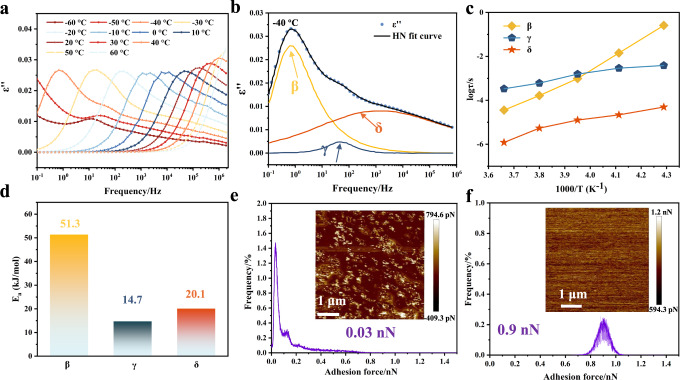
Characterization of activity for dynamic bonds at low temperature. **a** Dielectric loss *ε*′′ as a function of frequency for PDES–EAN-b from −60 to 60 °C. **b** Dielectric loss spectra of PDES–EAN-b fitted by a combination of three H–N equations at −40 °C. **c** The relaxation time as a function of temperature for multiple relaxation processes in PDES–EAN-b. **d** Activation energies (*E*_a_) of PDES–EAN-b for β-, γ-, and δ-relaxation. **e**, **f** AFM adhesion force images (5 μm × 5 μm) and the corresponding distributions of adhesion force for PDES–PEGDA and PDES–EAN-b samples, respectively.

The activation energy and multiplicity of secondary relaxations, including the movement of small motor units such as branch units, and end groups, are considered to be the keys to the self-healing ability of glassy polymers. To study the molecular dynamics of the obtained PDES–EAN sample, temperature-dependent broadband dielectric measurements were conducted (Fig. 3a). As shown in Fig. 3b, the dielectric spectrum of −40 °C can be fitted into three relaxation peaks by three terms analyses of Havriliak–Negami (H–N) function. They are assigned to β-relaxation (the fluctuation of end groups and branch units including –C_EAN_–OH, –COOH), γ-relaxation (the crank motion of the above groups), δ-relaxation (the twisting and swinging motion of side groups). The average relaxation times of the motions for these moieties can be extracted from the fitted H–N equation, and they are plotted as a function of temperature in Fig. 3c. The results show that the kinetic rate of δ and γ-relaxation is faster than that of β-relaxation, and all these three relaxation modes can take place under 0 °C^20^. It should be noted that the average relaxation time is in the range of 10^−6^–10^−2^ s at −20 °C, which prove that the terminals of EAN and functional units of the main chain are mobile in even low temperature. Subsequently, we employed the Arrhenius function to fit the active energies (*E*_a_) for the motions of different moieties. For PDES–EAN-b, the *E*_a_ values of the three relaxation processes are quite low, ranging from 14.7 to 51.3 kJ mol^−1^ (Fig. 3d). This explains why these relaxation modes can easily take place even in sub-zero conditions. As a result, the three secondary transformation processes lay the foundation for the materials to self-heal below *T*_g_, and even in low-temperature environments such as in subzero conditions. Dielectric loss data at different temperatures of PDES–EAN with various filler content was measured as shown in Supplementary Figs. 6–10 (Supplementary Data 4). The results demonstrate that when EAN is absent or introduced in relatively small proportions, the kinetic activation energy barriers of molecular segments and moieties increase at low temperatures, hindering the materials’ potential to be tough and subzero self-healable.

Moreover, quantitative studies of adhesion force for the surface layer were performed at 0 °C by AFM equipped with a silicon tip. Due to the presence of abundant hydroxyl groups on silicon tips, which are expected to build non-covalent bonds with moieties in PDES–EAN-b. The mappings and the corresponding distributions of the adhesion force of PDES–PEGDA and PDES–EAN-b are displayed in Fig. 3e, f, respectively. The average adhesion force for the surface layer of PDES–PEGDA is 0.03 nN, which is much lower than that of 0.9 nN for PDES–EAN-b. The superior adhesion performance of PDES–EAN-b can be attributed to the abundant free hydroxyl groups and loosely packed Fe^3+^ of EAN particles. These results also demonstrate the kinetic activity of hydrogen bonds and metal–hydroxyl interactions constructed between end groups and branch units at freezing temperatures. The extraordinary activity of dynamic bonds ameliorates the limitation of the self-healing process with a freezing polymer network, which makes it possible to achieve self-healing at sub-zero conditions. The adhesion energy at room temperature was evaluated via 180° peeling tests. As shown in Supplementary Fig. 11, the adhesion of PDES–EAN-b was noticeably higher than that of the control group, further confirming the multiple interactions constructed by EAN nanoparticles.

### Mechanical and self-healing properties

**Fig. 4:**
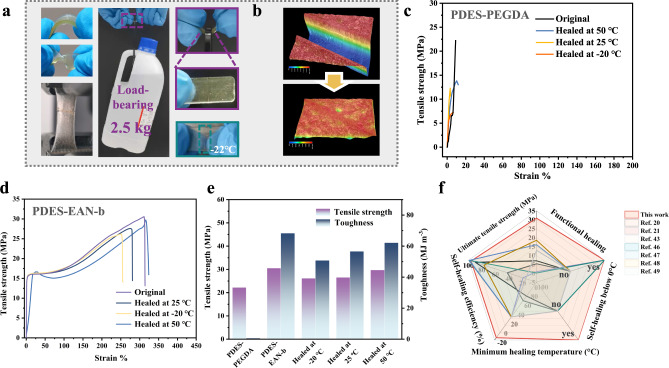
Mechanical and self-healing performance. **a** Photographs demonstrating the reliable self-healing ability of PDES–EAN-b when bent, twisted, stretched, and lift a weight. **b** Images of cut and healed PDES–EAN-b sample in the super depth of field three-dimensional microscope. **c** Tensile curves of the original and self-healed samples for PDES–PEGDA. **d** Tensile curves of the original and self-healed samples for PDES–EAN-b. **e** Comparison of tensile strength and toughness of PDES–PEGDA, original and self-healed PDES–EAN-b samples. **f** Comparison of ultimate tensile strength, functional healing ability, self-healing capacity below 0 °C, minimum healable temperature, and best self-healing efficiency of PDES–EAN-b with other self-healing materials.

The *T*_g_ for each composition as well as control of PDES–PEGDA was determined by dynamic mechanical analysis (DMA) and differential scanning calorimetric (DSC) (Supplementary Fig. 15). For the same sample, the *T*_g_ values obtained by DSC are ~6 °C lower than that of *T*_g_ values from DMA, which was primarily attributable to the different measurement mechanism of the two methods^41,42^. The *T*_g_ for the resulting PDES–EAN-b was determined to be 35.75 °C, confirming the glassy state of PDES–EAN-b at room temperature. Interestingly, the activity and mobility of dynamic bonds at low temperatures endow the materials with extraordinary self-healing ability, although PDES–EAN-b is a mechanically robust polymer. As shown in Fig. 4a, the cut and re-contacted samples can easily bear bending, torsion, and tensile loads after self-healing at −20 °C for 6 h. Meanwhile, the obtained self-healed sample can easily lift 25,000 times its own weight without any damage and growth of the crack. After removing this sheet from the fixtures, we found that the healed sheet had a little dimensional extension. Since the motion activity of molecular groups is greatly affected by temperature, it exhibits a significant increase as the ambient temperature rises. The rapid self-healing process at room temperature was observed by the ultra-depth microscope. As shown in Fig. 4b, the scar at damage became almost invisible after just 5 min of contact, which is rarely achieved in glassy polymers.

Figure 4c shows that the PDES–PEGDA sample possesses a strength of 22.2 MPa, which is significantly stronger than most of the DES-based materials (usually 0.1–5 MPa)^43–45^ due to the rigid anhydride ring of MAH in the polymer chain. Thermogravimetric analysis (TGA) shows that temperatures of PDES–PEGDA and PDES–EAN-b at 10% weight loss are both above 200 °C (Supplementary Fig. 16), demonstrating their excellent thermal stability. The MAH microdomain acts as a physical cross-linking point in the polymer molecular chain, limiting the slippage of the molecular chain and improving strength. After introducing EAN, the tensile strength and toughness of the material have been increased by 1.4 and 95.2 times for PDES–EAN-b, respectively. This is largely derived from the high density of synergistic dynamic bonds and physical confinement of nanospheres. It is worth noting that the healing efficiency of PDES–EAN-b specimen after healing at −20 °C is up to remarkably 85.7% in strength recovery, shown in Fig. 4d, e, proving the impressive self-healing ability. With the increase in temperature, the self-healing efficiency is further improved (86.9% at 25 °C, 97.3% at 50 °C). It is probably because the movement of PDES segments and the dissociation–reconstruction process of dynamic bonds have been accelerated as the ambient temperature rises. In addition, the mechanical properties and self-healing ability of samples with different EAN content were also measured by tensile tests, as shown in Supplementary Fig. 12 (Supplementary Data 5). The results show that when the amount of EAN is small, the elongation at the break of the material is reduced and the self-healing ability is insufficient. This further confirms that the active fracture and reconstruction of a dynamic bond network built by EAN improved the toughness and enable the material to heal after damage. As the substitution amount of EAN reaches 0.06 wt%, the mechanical strength of the material evidently decreases, which is probably due to the aggregation of EAN in the PDES matrix, and the excess EAN may interfere with the photopolymerization of DES. Notably, PDES–EAN-b shows the optimal mechanical and self-healing properties, which exceed most of the reported self-healing polymers^20,21,43,46–49^, shown in Fig. 4f (details in Supplementary Table 1). This makes it attractive for applications in e-skins aspects. We further explored the self-healing performance of PDES–EAN-b at −30 and −40 °C, as shown in Supplementary Fig. 14. The results show that the sample can still self-heal at lower temperatures, but the efficiency of self-healing declines.

### Wide temperature range strain-sensing and subzero healing abilities

**Fig. 5:**
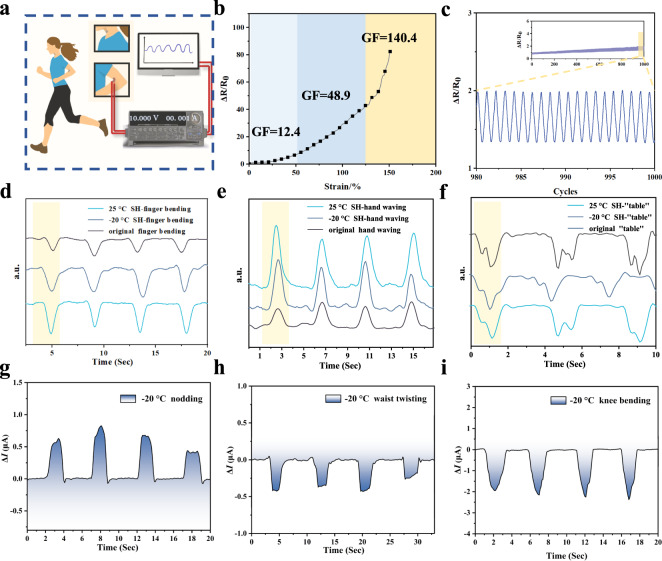
Strain-sensing performance. **a** Schematic illustration of the real-time sensing platform based on PDES–EAN sensor, a SourceMeter, and a computer. **b** Sensitivity test with 0–150% strain. The slope of linear fitting corresponds to the GFs in the range of strain from 0% to 50%, 50% to 130%, and 130% to 150%, respectively. **c** Reproducibility tests for over 1000 stretching cycles at 50% strain. Various sensing signals when the volunteer is performing different actions equipped with the original/self-healed sensor at 25 °C: **d** finger bending, **e** hand waving, **f** vocal-cord vibration when pronouncing “table”, and with the self-healed sensor at −20 °C: **g** nodding, **h** waist twisting, and **i** knee bending.

Based on the free ions’ directional migration ability provided by ChCl, the ionic conductive polymer can work as a resistive sensor under an applied external electric field. As shown in Fig. 5a, the obtained sample is expected to act as an e-skin to capture human motions and even speech recognition. The high adhesiveness produced by the multiple hydrogen bonds allows the material to stick tightly to the skin. By connecting to a circuit, the signals generated by motions can be collected by a digital multimeter or a Source Meter. And the signals will be displayed on the computer/smartphone in real-time, so as to effectively monitor and analyze outdoor sports and indoor activities. To quantify the sensitivity of stretching-related resistance changes, the gauge factor (GF) was obtained by calculating the slope of the relative resistance-strain curve. Notably, the GF values range from 12.4 to 140.4 while the sensor was stretched, demonstrating ultra-high sensitivity in the field of DES-based materials, as shown in Fig. 5b. Moreover, the PDES–EAN-b sample shows great repeatability in the stretch-release experiments as a strain-sensing electronic. During the process of over 1000 cycles of rapid reproducibility test, the output current peak pattern remains stable even throughout the last 20 cycles shown in Fig. 5c. Here, the flexible samples have been attached to several body parts of a volunteer including knuckles, wrists, and Adam’s apples to achieve movement monitoring. Figure 5d–f shows that the original sample and the self-healed sample exhibit consistent electrical peak output for the same motion, indicating the effective recovery of the ionic conductive pathway during the healing process, which further confirms its potential to be used as e-skins. More motion and voice monitoring tests are shown in Supplementary Figs. 18–21. We further tested the motion detection ability of the sample at subzero temperatures. The data of temperature-dependent conductivity are included in Supplementary Fig. 17. In order to minimize the influence of temperature change and instability, the material was attached to the relevant parts of the wooden doll during the experiment, and it was frozen in the freezer below −20 °C for 2 h before starting the test. The results show that the sample is sensitive and specific to actions of nodding, twisting the waist, and bending a knee in Fig. 5g–i (Supplementary Data 6), demonstrating excellent low-temperature sensing capability, especially in the field of ionic conductive materials.

Combined with its cryogenic self-healing ability, the material’s potential applications in wearable electronics could be greatly expanded. For example, in the Winter Olympics, sensing devices were extensively applied and especially play indispensable roles in monitoring athletes’ technical movement, physiological information, etc., which standardizes athletes’ technology, ensures safety, and improves competitive performance. The majority of sensing materials based on conventional elastomers and hydrogels, especially those with *T*_g_ above 0 °C, suffer from rigid and brittle defects at low temperatures^17,50–53^. For instance, polyurethane with multiple hydrogen bonds (PU/CD-SG, *T*_g_ < −50 °C)^40^, PDES–PEGDA, and highly stretchable PDES (CNCs-PDES, *T*_g_ ≈ 4 °C)^24^, all exhibit good mechanical properties but are difficult to recover from damage at low temperatures (Supplementary Fig. 13 and Movies 1–3). Upon suffering from damages at low temperatures, healing is expected to be accomplished through the strategy of dense dynamic networks with rapid secondary relaxations reported in this work.

## Discussion

In summary, we have proposed here a glassy polymer with the subzero self-healing ability and strong mechanical properties taking advantage of the metal-doped polyphenol nanosphere. The unique polyphenol assembly induced by Fe^3+^ contains a large number of non-covalent binding sites distributed on the surface, which endows the construction of hydrogen bonds and metal coordination bonds with the comb-like arranged branch units (–COOH) from PDES. The combination of rapid secondary relaxations with low activation energy barriers and multiple dynamic cross-linking networks provides a promising method to ameliorate the limited self-healing ability of glassy polymers. The obtained PDES–EAN-b nanocomposite exhibits outstanding tensile strength and excellent healing efficiencies even at −20 °C. In addition, the ionic conductivity derived from ChCl allows the material to be used as a strain sensor and exhibits favorable functional reliability over a wide temperature range. It is important to note that such nano-assemblies with active end groups are widely applicable and not limited to spheres, but worth exploring any morphologies that are beneficial to enhance the activity of dynamic bonds, such as sea urchin-like, coiled ribbons-like shapes.

## Methods

### Materials

Acrylic acid (AA, AR, Shanghai Macklin Biochemical Co., Ltd.), maleic anhydride (MA, AR, Shanghai Macklin Biochemical Co., Ltd.), choline chloride (ChCl, 99%, Shanghai Titan Scientific Co., Ltd.), tannic acid (TA, AR, Sinopharm Chemical Reagent Co., Ltd.), Iron trichloride (FeCl_3_·6H_2_O, AR, Damao Chemical Reagent Factory), 2-hydroxy-4-(2-hydroxyethoxy)−2-methylpropiophenone (photoinitiator 2959, ≥98%, Shanghai Titan Scientific Co., Ltd.), and poly (ethylene glycol) diacrylate (PEG(200)-DA, Shanghai Macklin Biochemical Co., Ltd.) were used as received. The water used in all experiments was deionized and ultrafiltered to 18.2 MΩ cm using the Ulupure ultrapure water system.

### Sonication and assembly of TA

The assembly process was performed using an ultrasonic cell crusher, which is equipped with adjustable power from 0 to 1500 W. Driven by a high frequency of ultrasound, the cavitation bubbles were produced in liquid, which can be used as micro-reactors to give chemical effects such as inducing the homolysis of water to generate radicals. In brief, 2.5 mM TA aqueous solution was prepared in a glass vial and was treated with 5.5 W cm^−3^ ultrasound for 10 min. Note that the temperature was kept at 0 °C with an ice water bath during the whole sonication experiment. After that, 18.4 mM FeCl_3_ aqueous solution was added to the pretreatment solution through the above steps in a 1:3 mole ratio for another 5 min of ultrasonic treatment to complete the assembly of EA nanospheres (EAN). Then, the final suspension was freeze-dried and collected for further use. Specifically, the specific size of TA nanoparticles assembled by ultrasound is positively correlated with the concentration of TA aqueous solution and the ultrasound time. In order to more conveniently observe the assembly process of TA in the absence of Fe^3+^, we increased the TA concentration by 0.2 times and extended the ultrasound time to 30 min in the control group, shown in Fig. 2a.

### Synthesis of photopolymerizable deep eutectic solvent (PDES)

The reaction precursor was prepared by mixing MAH, ChCl, and AA (molar ratio MAH:ChCl:AA = 1:2:6) in a closed flask. Then, the mixtures were stirred and heated at 90 °C for 2 h when the colorless homogeneous solutions were obtained. The formed MAH/ChCl-AA/ChCl type PDES was then stored in a vacuum desiccator with silica gel for further use.

### Photopolymerization of PDES–EAN

The monomers (MAH/ChCl-AA/ChCl type PDES), 0.1 mol% photoinitiators 2959, and 0.1 mol% crosslinker PEG (200) DA to monomers were mixed. 0.02, 0.04, and 0.06 wt% EAN powder to monomers was added into the mixtures and dispersed with bath sonication treatment until uniform mixtures were formed, denoted as PDES–EAN-a, PDES–EAN-b, PDES–EAN-c, respectively. The control sample (denoted as PDES–PEGDA) was prepared using MAH/ChCl-AA/ChCl as monomers with the same proportion of photoinitiator 2959 and 0.1 mol% crosslinker PEG (200) DA. The resulting liquids were poured into a polytetrafluoroethylene mold and polymerized by 360 nm UV irradiation for 1 h.

### Characterization

TEM was performed on a transmission electron microscope (JEOL JEM-100CX, Japan). XPS was recorded on an ESCALab220i-XL electron spectrometer from VG Scientific (USA) using 300 W Al Ka radiation. Laser confocal microscopy Raman spectroscopy (HORIBA, HR Evolution, Japan) with a 785 nm laser line was performed to characterize metal–ligand coordination. AFM measurements were performed on a Bruker Bio-FastScan AFM using silicon tips (OMCLAC160TS-R3, Olympus) in the PeakForce quantitative nanomechanical property mapping mode. DMA measurements were performed on a TA Instrument (Q800, USA) in strain-controlled mode with a frequency of 1 Hz and a heating rate of 5 °C min^−1^ in the range of −40 to 80 °C. DSC analysis was generated on a TA Instrument (DSC Q1000, USA) with a liquid nitrogen cooling system. Samples were heated at a rate of 10 °C min^−1^ from −30 to 120 °C. TGA was carried out using TA Instruments (Q500, USA) at a heating rate of 10 °C min^−1^ from 30 to 650 °C under a nitrogen atmosphere. Super deep scene 3D microscope (ZEISS Smartzoom 5, Germany) was performed to detect the fractured surface morphology of the cut and self-healed sample. The sensor was connected to a conductive circuit with Keithley 2601B source meter (USA) which can measure the current signal in real-time to test the sensitivity and electrical self-healing performance. 180° peeling tests were carried out using Instron 5560 (USA). Before the test, two rectangular samples of 15 × 1 cm^2^ were laminated on each other and preloaded by 800 g weight for 30 min. The samples were then delaminated at a rate of 50 mm min^−1^.

### Binding energy simulation

The molecular dynamic simulation was performed under a universal force field in Material Studio 2018. Firstly, molecular models of TA, EA, and the PDES with 20° of polymerization (repeating units: -AA-AA-AA-AA-AA-AA-MAH-) were constructed and geometrically optimized using the Forcite module. Next, the amorphous PDES cells were constructed by a predigested method. According to the principle of equal volume comparison, multiple EA molecules were loaded into an EA group, where the content of catechol was the same as that of a TA molecule. Subsequently, a TA molecule and an EA group were loaded into PDES cells respectively, and then geometrical optimizations were performed again. After each component was modeled, the overall system with initial energy minimization was subsequently built. To further obtain the optimized conformation, the isothermal–isochoric molecular dynamic simulation of PDES-TA composite and PDES-EAN composite models was conducted at 600 K. Then, the binding energy can be calculated based on the final conformation models.

### Temperature-dependent FTIR spectroscopy

A Nicolet iS50 Fourier transforms spectrometer (USA) equipped with a deuterated triglycine sulfate detector was used for the temperature-dependent FTIR experiments in the range of 4000–1000 cm^−1^. The obtained PDES-EAN sample was firstly sandwiched between two CaF_2_ windows and then was heated from 25 to 180 °C at 5 °C min^−1^. Each FTIR spectrum was obtained from 20 scans with a resolution of 4 cm^−1^. Note that the samples were protected by high-purity nitrogen gas (200 mL min^−1^) during the experiment. Then, the perturbation-correlation moving-window two-dimensional (PCMW2D) correlation FTIR spectra were processed, calculated, and plotted using 2DCS software. To obtain credible results, the baseline correction was performed before analysis.

### Broadband dielectric measurements and analyses

Broadband dielectric measurements were performed on a Novocontrol Concept 50 system with Quatro Cryosystem temperature control and an Alpha impedance analyzer. The circular film with 20 mm in diameter and 1 mm thickness was placed into two parallel electrodes. Frequency sweep mode used the frequency range of 10^−1^–10^7^ Hz at each temperature from −60 to 60 °C with 10 °C intervals.

In order to study the local dynamic characteristics of PDES–EAN sample, especially below *T*_g_, Havriliak and Negami (H–N) function was employed to analyze the dielectric spectra. In this model, the dielectric complex permittivity (ε*) data as a function of frequency can be expressed by the following formula:

2 $${\varepsilon }^{*}={\varepsilon }_{{{{{{\rm{\infty }}}}}}}+\frac{\varDelta \varepsilon }{{\left[1+{\left({i\omega \tau \tau }_{{HN}}\right)}^{\alpha }\right]}^{\beta }}$$

where *τ*_HN_ is the characteristic relaxation time, Δ*ε* is the relaxation strength and *ε*_∞_ is the unrelaxed values of the dielectric constant. The *α* and *β* parameters (*α* < 0, *αβ* ≤ 1) define the symmetrical and asymmetrical broadening of the loss peak, respectively.

The relationship between average relaxation time (*τ*_max_) and *τ*_HN_ can be given by the following equation:

3 $${\tau }_{{\rm {m{ax}}}}={\tau }_{{{\rm {HN}}}}{\left[\sin \frac{\pi \alpha \beta }{2\left(1+\beta \right)}\right]}^{-\frac{1}{\alpha }}{\left[\sin \frac{\pi \alpha }{2\left(1+\beta \right)}\right]}^{-\frac{1}{\alpha }};\,\,{{f}}_{\max }=\frac{1}{2\pi {\tau }_{{\rm {m{ax}}}}}$$

where *f*_max_ is the frequency at which *ε*′′ passes through the maximum value.

The relation between temperature and the average relaxation time can be described using Arrhenius functions:

4 $${\tau }_{\max }={\tau }_{0}\exp \left(\frac{{E}_{{\rm {a}}}}{{RT}}\right)$$

where *E*_a_ is the activation energy and *τ*_0_ is a constant. The Arrhenius fits were carried out at these temperatures where the patterns of all relaxation peaks (*α*, *β*, *γ*) are complete.

### Mechanical and self-healing tests

The mechanical properties of PDES–EAN samples were characterized by using Instron 5560 (USA) with a 1000 N load cell. The samples have a central part of 15 mm in length, 2 mm in width, and 0.6–0.9 mm in thickness. Mechanical tensile stress and mechanical self-healing property tests were performed at room temperature with a strain rate of 50 mm min^−1^. Samples used to test self-healing properties were prepared by cutting in half first with a blade and bringing the two fresh sections into contact immediately. Damaged samples were given 1 h to heal at 25 and 50 °C, and 6 h to heal at −20, −30, −40 °C, respectively. Prior to tensile tests, the 50 °C self-healed samples were cooled to room temperature for two hours. The mechanical healing efficiency (*η*) is defined as the proportion of strength restored relative to the original one.

## Supplementary information

Supplementary Information

Description of Additional Supplementary Files

Supplementary Data 1

Supplementary Data 2

Supplementary Data 3

Supplementary Data 4

Supplementary Data 5

Supplementary Data 6

Supplementary Movie 1

Supplementary Movie 2

Supplementary Movie 3

## Data Availability

The data that support the findings of this study are available from the corresponding author upon request. The data generated in this study are provided in the Supplementary Information/Source Data file. Source data are provided with this paper.

## Source data

Source Data
