# Supplementary material for: Ultrarobust subzero healable materials enabled by polyphenol nano-assemblies
Source: Nat Commun. 2023 Feb 13;14:814. doi: 10.1038/s41467-023-36461-9 (PMC9925762; doi:10.1038/s41467-023-36461-9)
Supplement: Supplementary file 1 — Supplementary Information [file 41467_2023_36461_MOESM1_ESM.pdf]

Supplementary Information

Ultrarobust subzero healable materials enabled by polyphenol nano-assemblies

Nan Wang[1], Xin Yang[1] & Xinxing Zhang[1*]

[1] State Key Laboratory of Polymer Materials Engineering, Polymer Research Institute, Sichuan University, Chengdu 610065, China
Correspondence and requests for materials should be addressed to X.Z. (email: xxzwwh@scu.edu.cn)

Supplementary Figures:

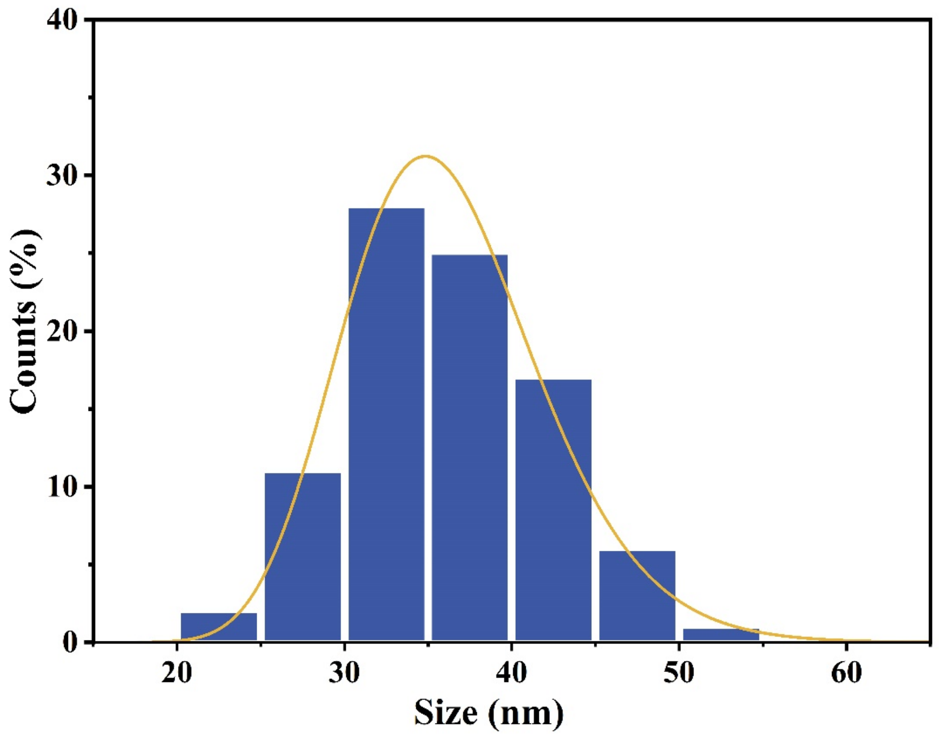

Supplementary Figure 1. Particle size distribution of EAN nanospheres.

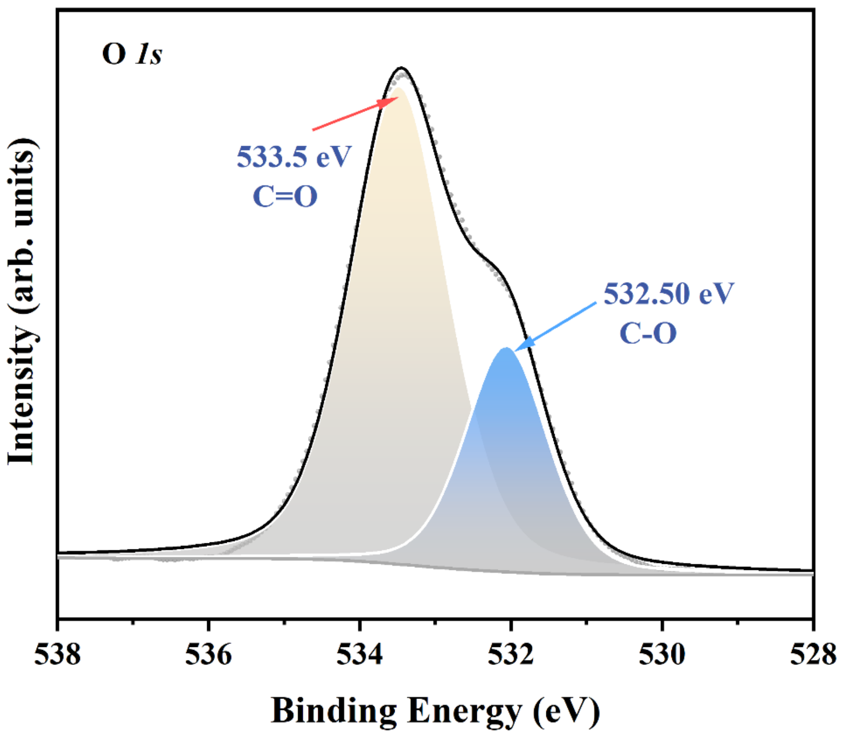

Supplementary Figure 2. The O 1s segment in the XPS spectra of EAN.

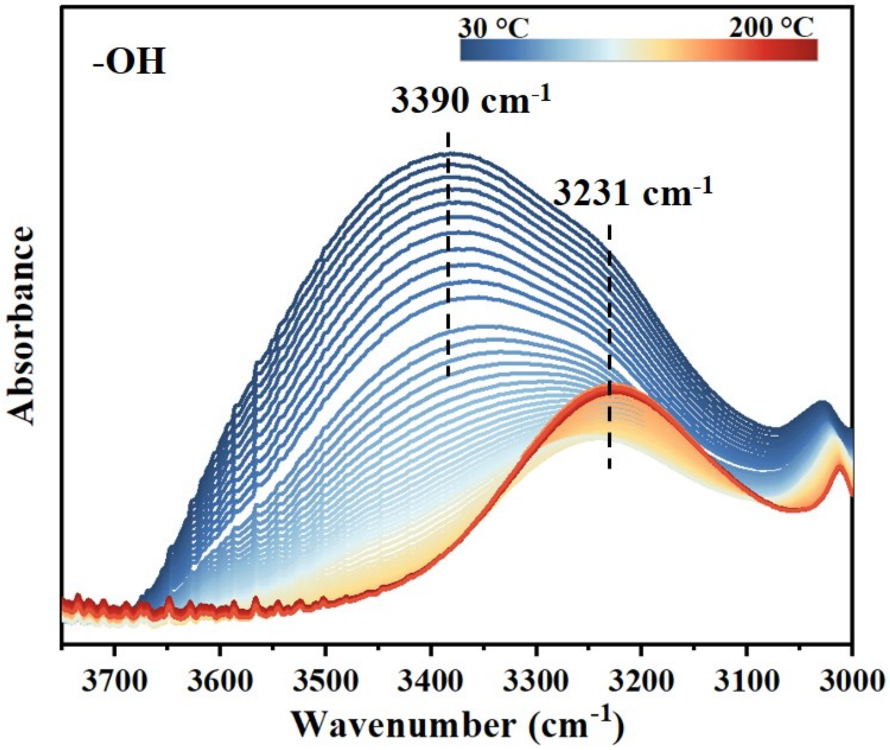

Supplementary Figure 3. FT-IR spectra of EAN.

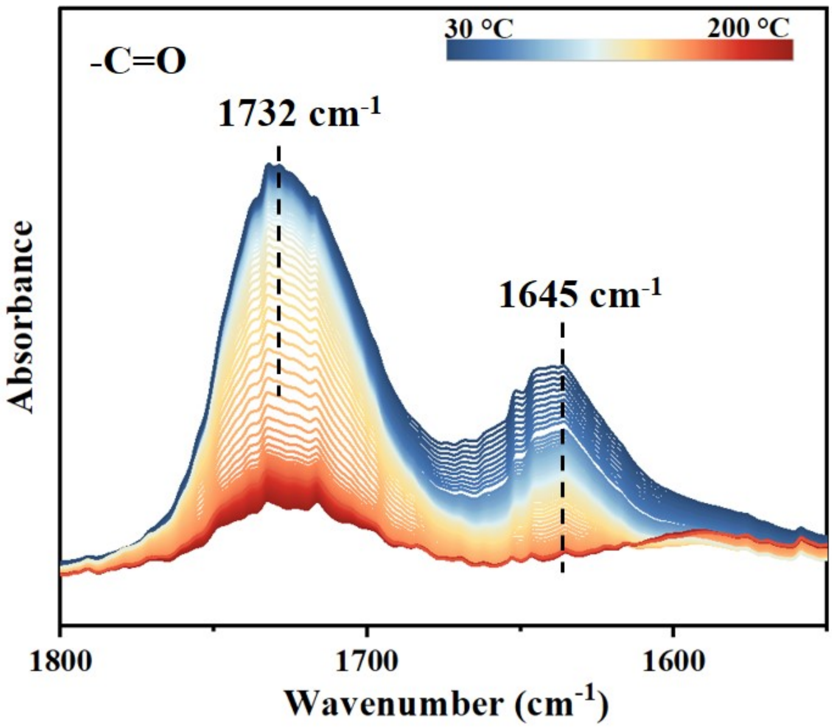

Supplementary Figure 4. FT-IR spectra of EAN.

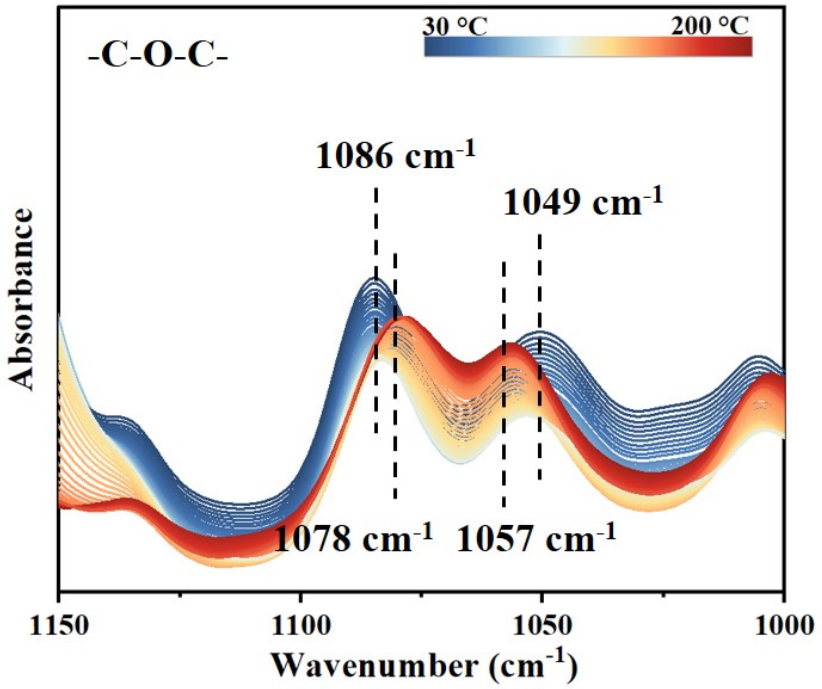

Supplementary Figure 5. FT-IR spectra of EAN.

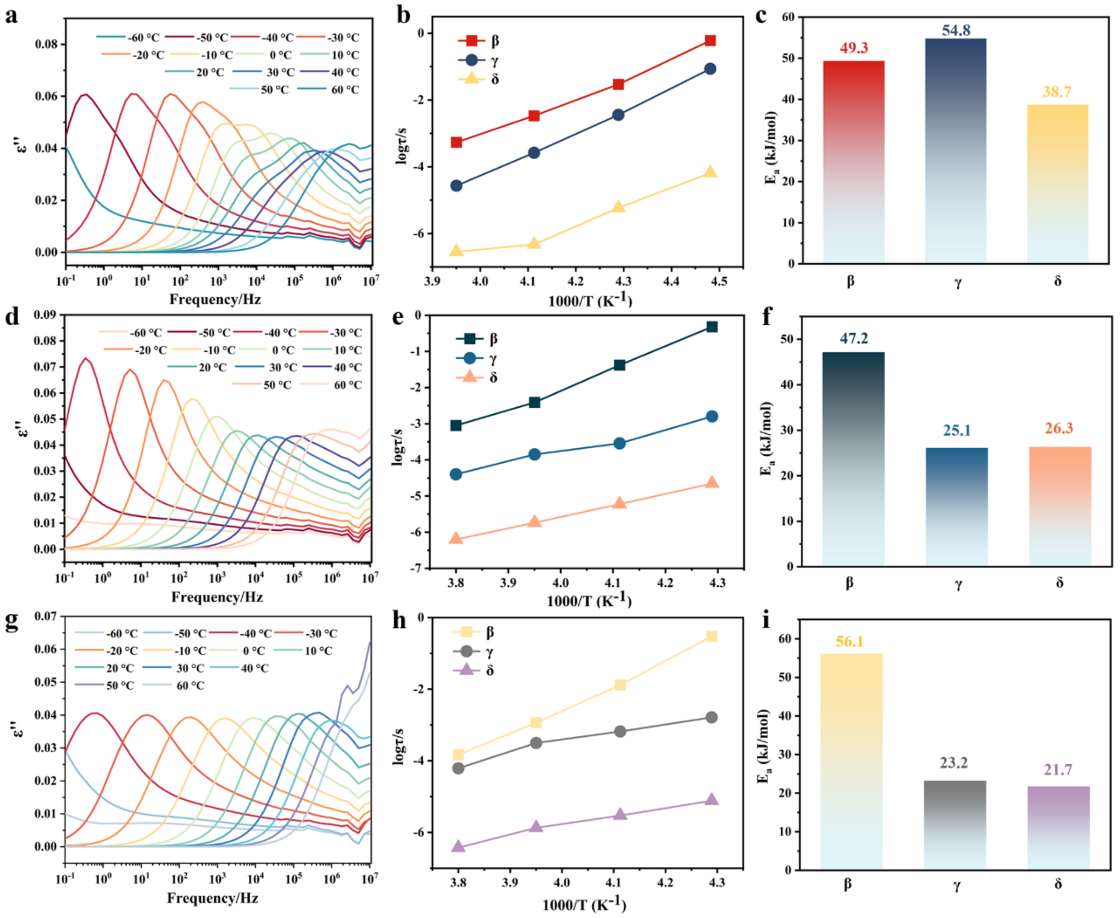

Supplementary Figure 6. Dielectric loss (ε'') as a function of frequency for PDES-PEGDA **(a)**, PDES-EAN-a **(d)**, PDES-EAN-c **(g)** ranged from -60 to 60 °C; The relaxation time as a function of temperature of different moieties for PDES-PEGDA **(b)**, PDES-EAN-a **(e)**, PDES-EAN-c **(h)**; Active energies (E_a) of PDES-PEGDA **(c)**, PDES-EAN-a **(f)**, PDES-EAN-c **(i)** for β-, γ-, and δ-relaxation.

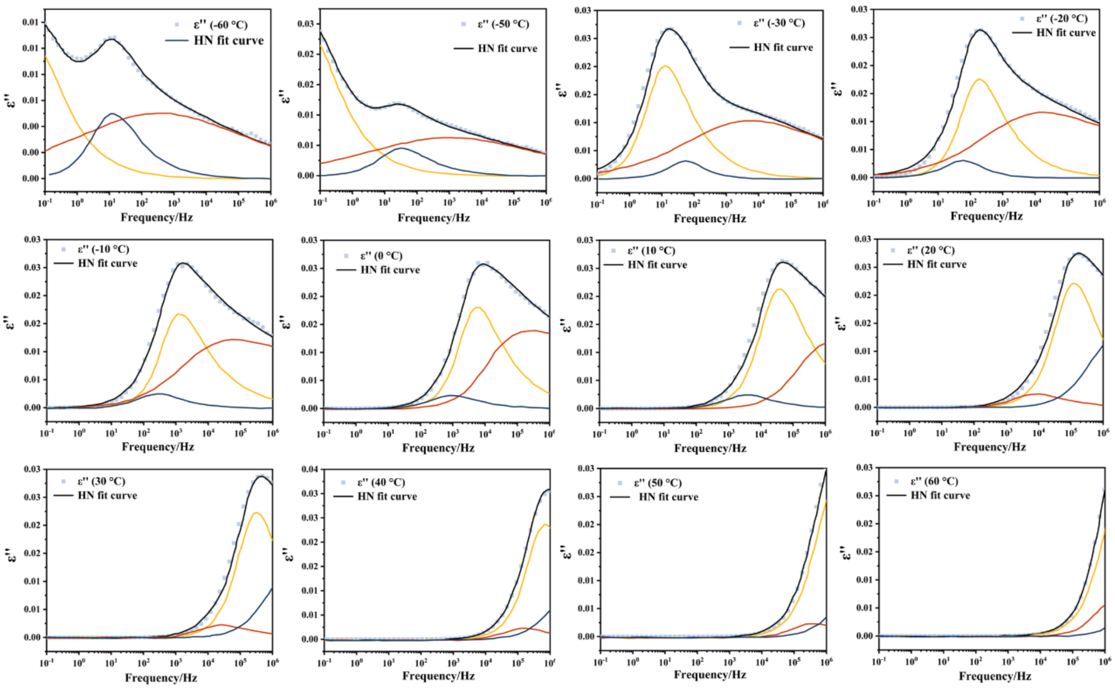

Supplementary Figure 7. Dielectric loss spectra of PDES-EAN-b fitted by a combination of three H-N equations from -60 to 60 °C.

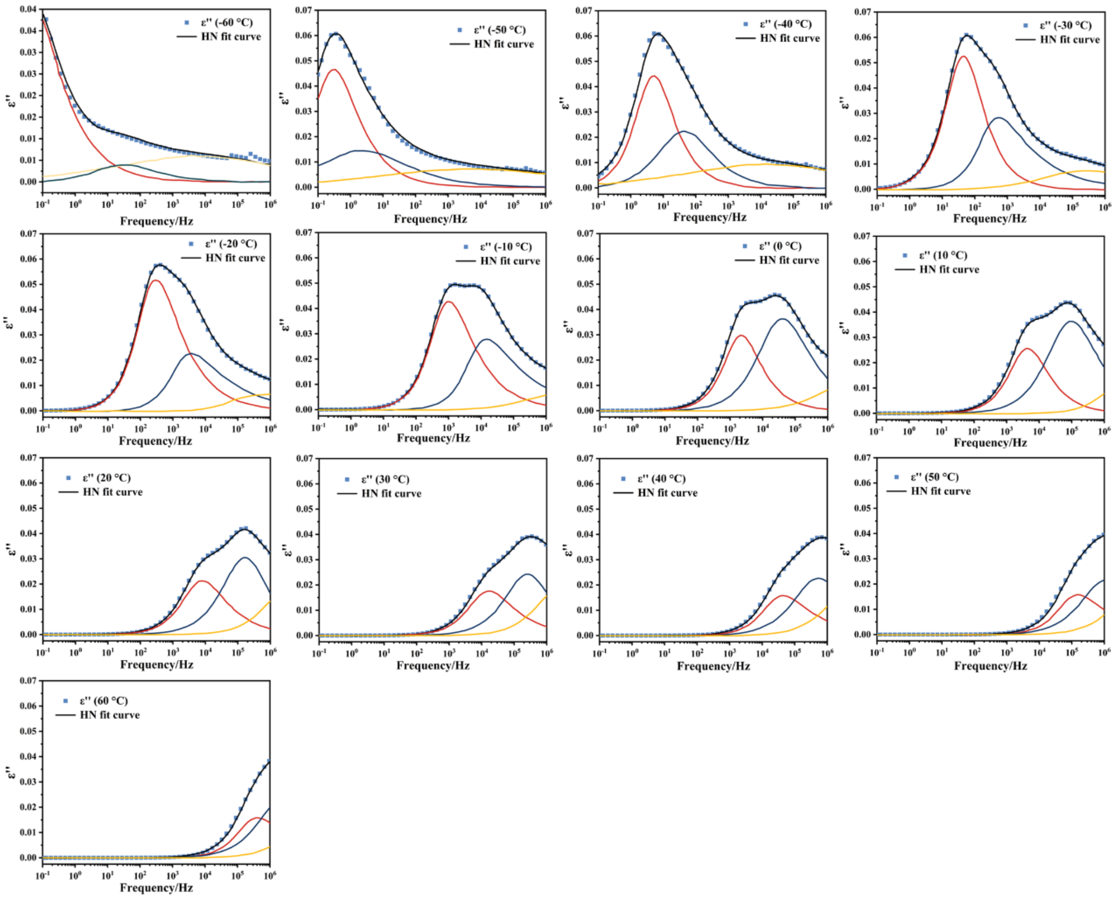

Supplementary Figure 8. Dielectric loss spectra of PDES-PEGDA fitted by a combination of three H-N equations from -60 to 60 °C.

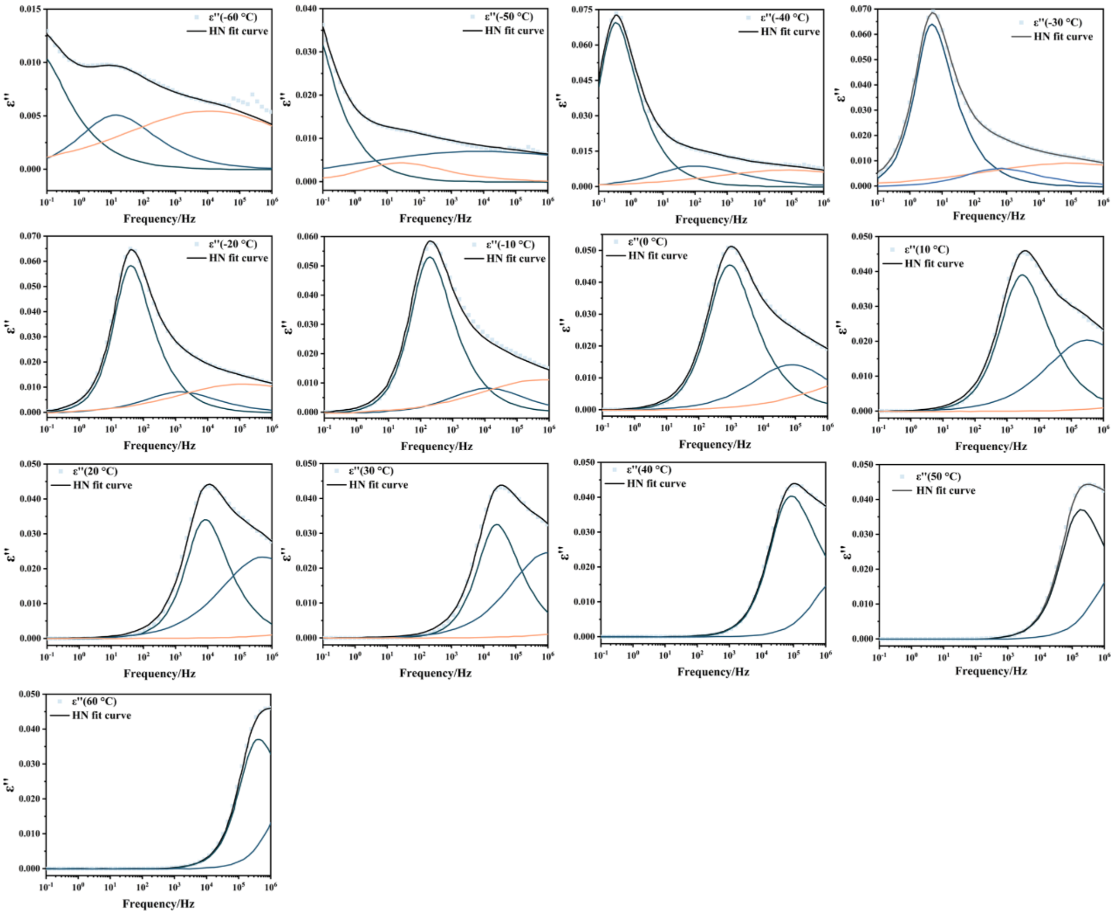

Supplementary Figure 9. Dielectric loss spectra of PDES-EAN-a fitted by a combination of three H-N equations from -60 to 60 °C.

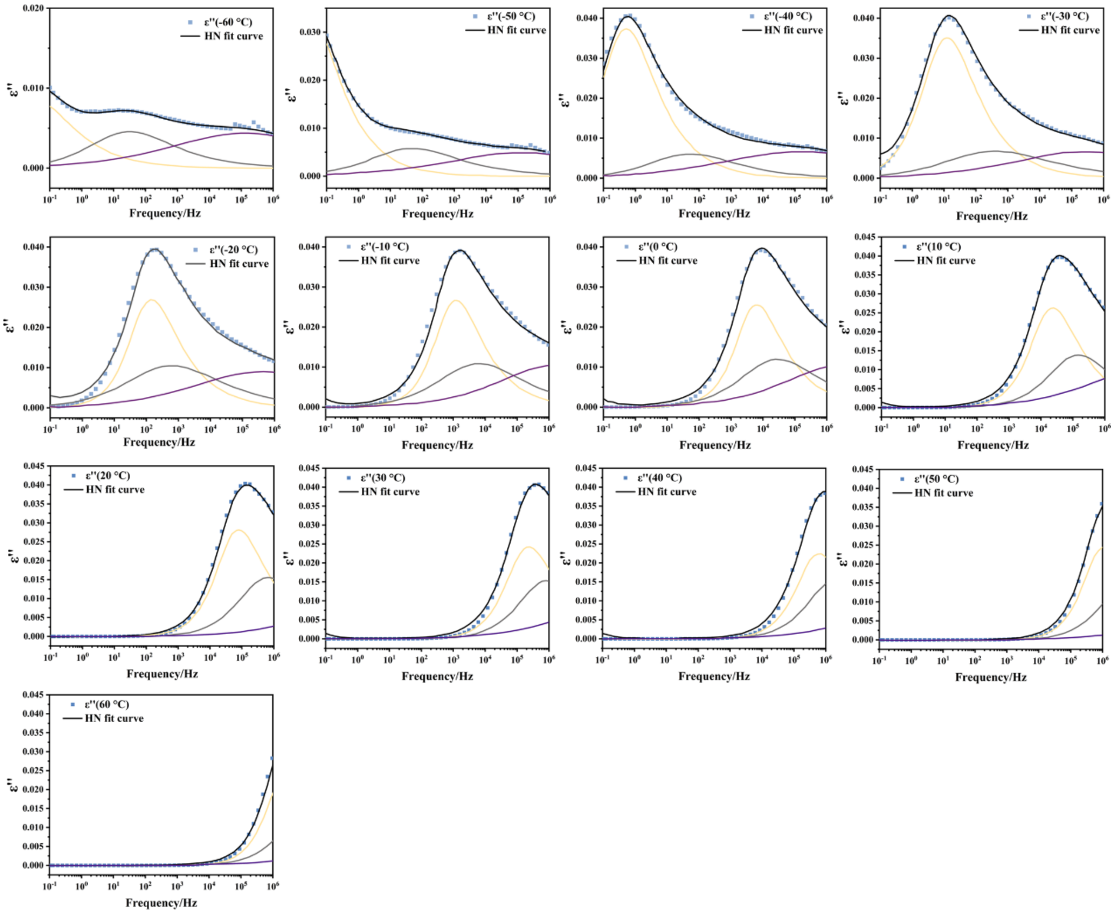

Supplementary Figure 10. Dielectric loss spectra of PDES-EAN-c fitted by a combination of three H-N equations from -60 to 60 °C.

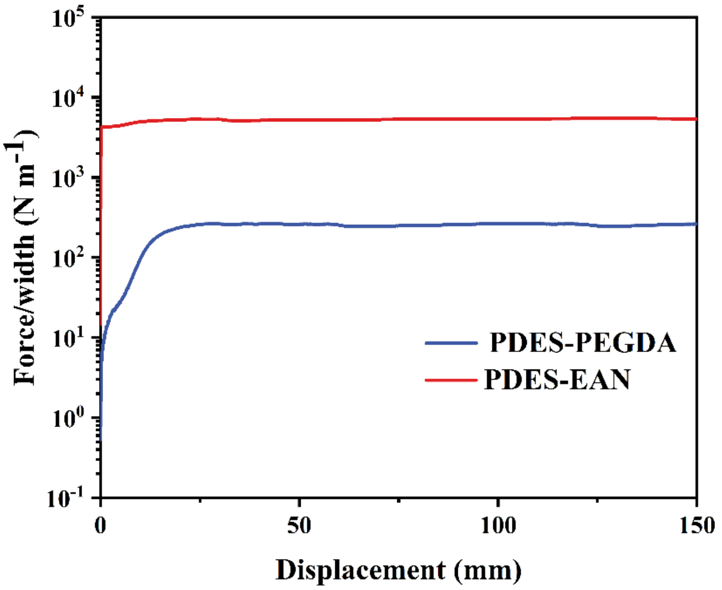

Supplementary Figure 11. 180 degree peeling curves of PDES-PEGDA and PDES-EAN-b.

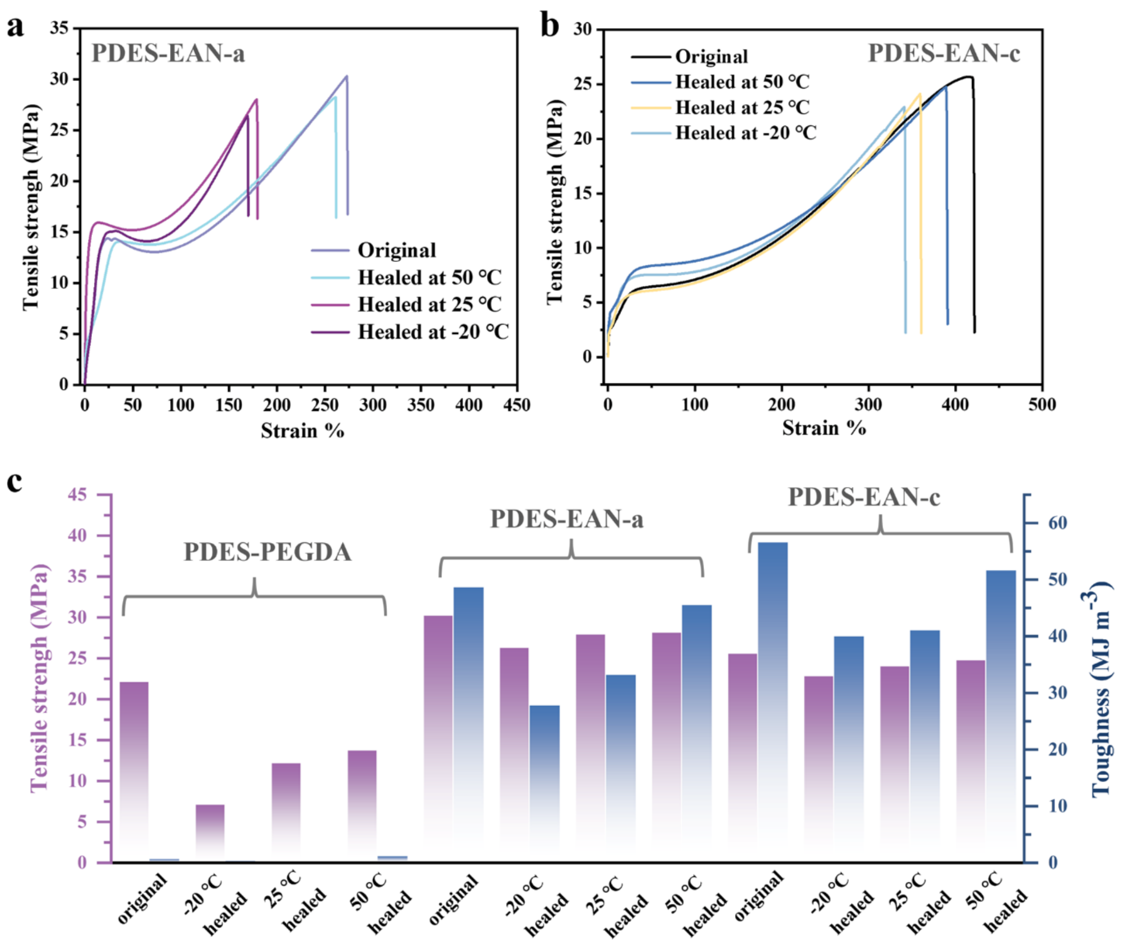

Supplementary Figure 12. Tensile curves of the original and self-healed samples for PDES-EAN-a **(a)** and PDES-EAN-c **(b)**; Comparison of tensile strength and toughness of original and self-healed PDES-PEGDA, PDES-EAN-a, PDES-EAN-c samples **(c)**.

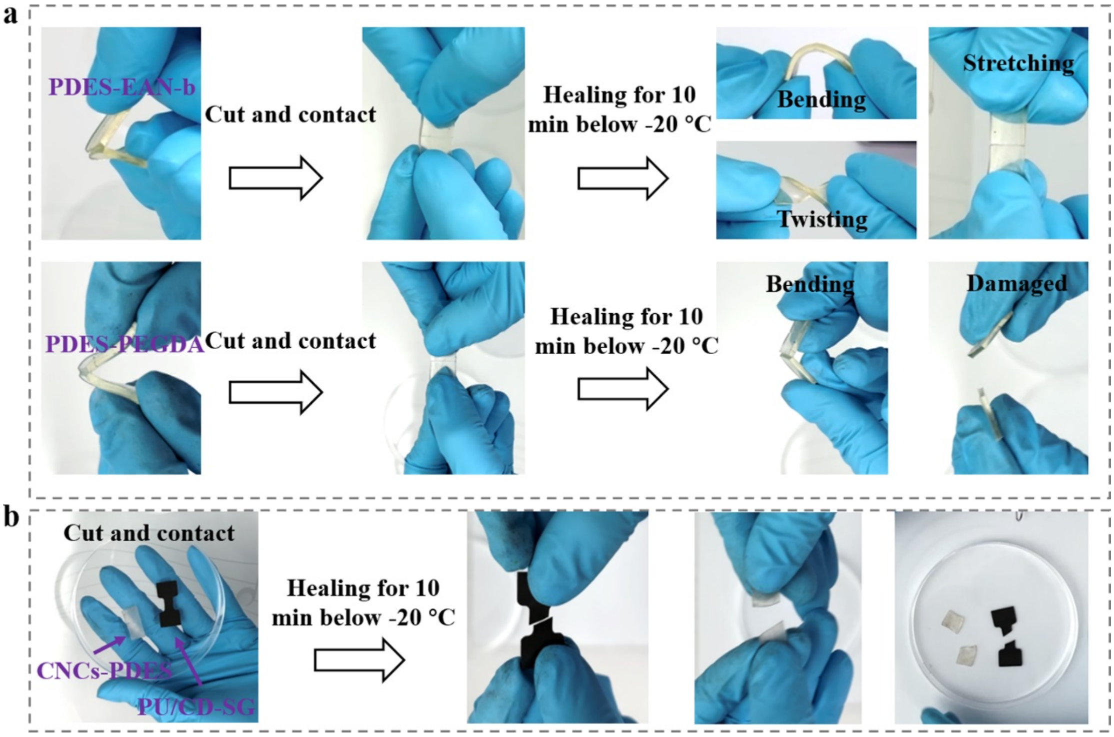

Supplementary Figure 13. Photographs demonstrating the subzero self-healing performance of PDES-EAN-b and PDES-PEGDA **(a)**, highly stretchable PDES (CNCs-PDES, T_g <-50 °C) and polyurethane with multiple hydrogen bonds (PU/CD-SG, T_g ≈4 °C) **(b)**.

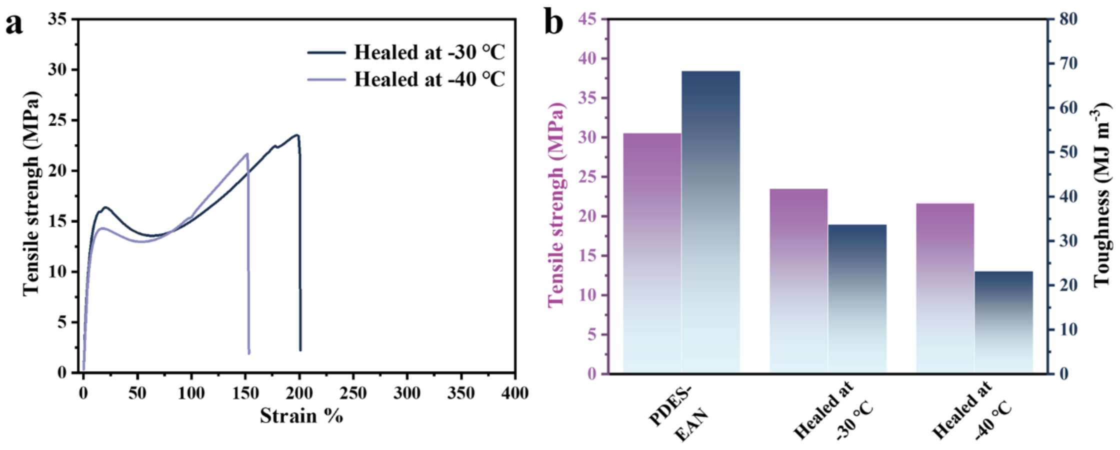

Supplementary Figure 14. Tensile curves of PDES-EAN-b samples after self-healing at -30 and -40 ℃ **(a)**; Comparison of tensile strength and toughness of original and self-healed PDES-EAN-b samples **(b)**.

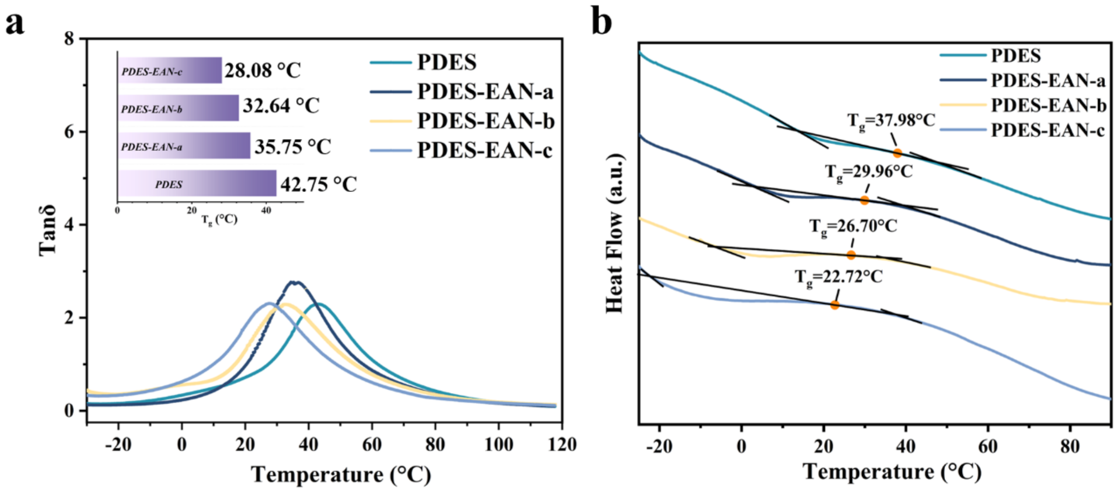

Supplementary Figure 15. The DMA **(a)** and DSC **(b)** curves of samples with multiple different EAN: PDES compositions.

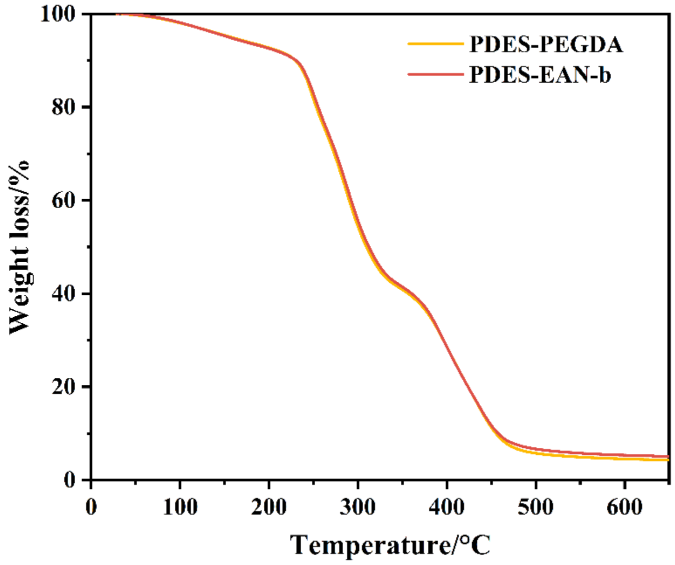

Supplementary Figure 16. TG curves of PDES-PEGDA and PDES-EAN samples upon heating from 30 °C to 650 °C at a rate of 10 °C /min.

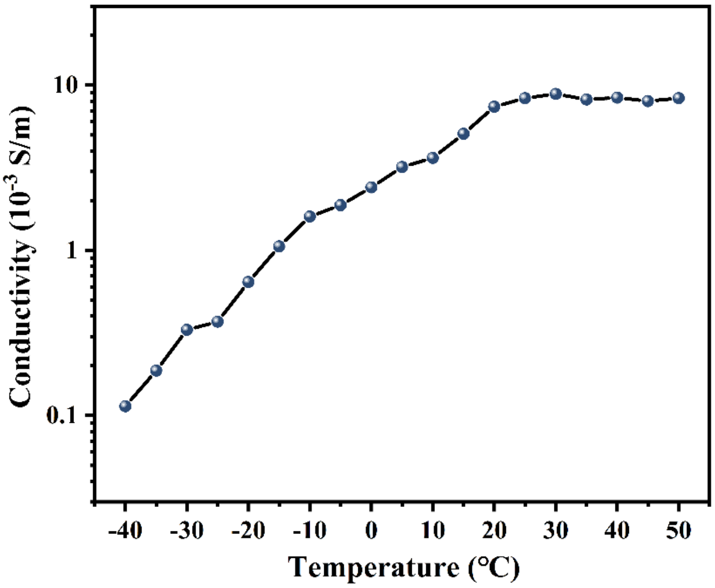

Supplementary Figure 17. The temperature-dependent conductivity curves of PDES-EAN-b.

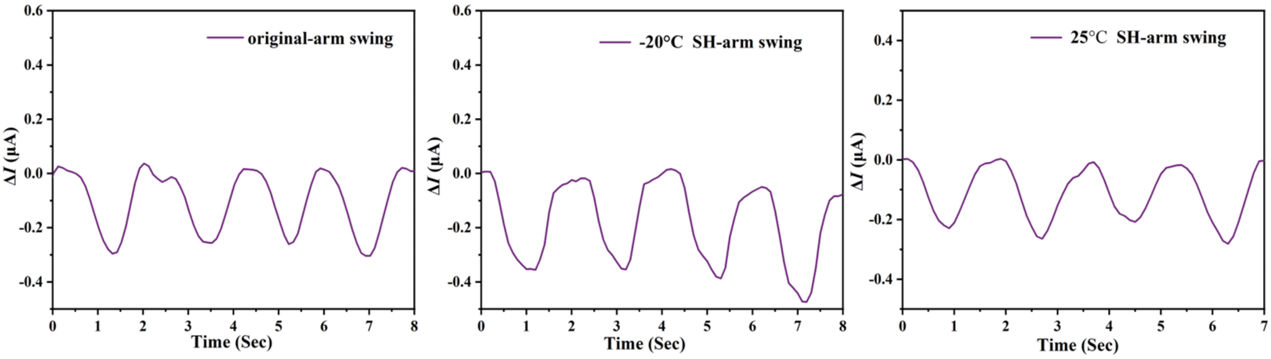

Supplementary Figure 18. Current responses of the original, -20 °C self-healed and 25 °C self-healed sensors in body movements.

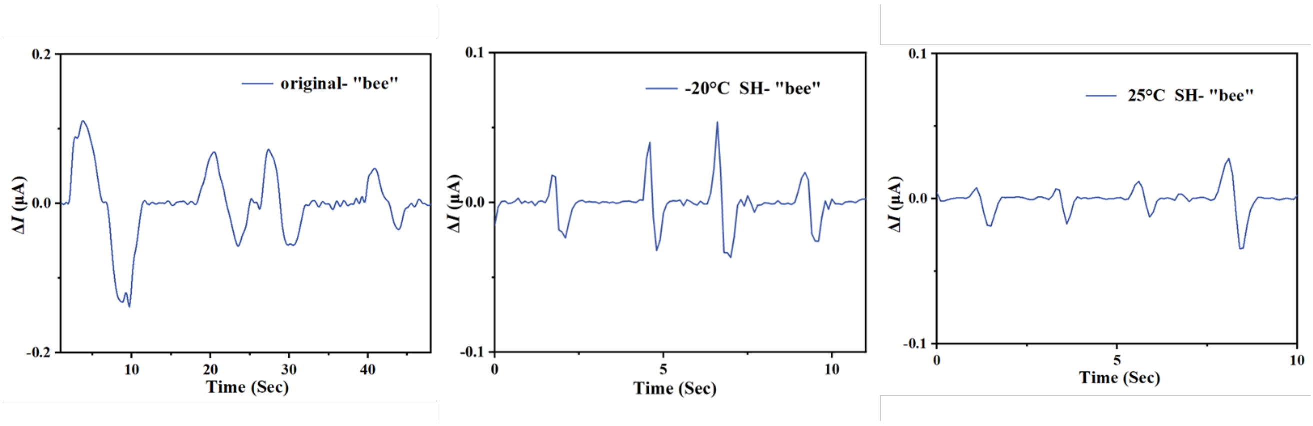

Supplementary Figure 19. Current responses of the original, -20 °C self-healed and 25 °C self-healed sensors in vocal-cord vibrations.

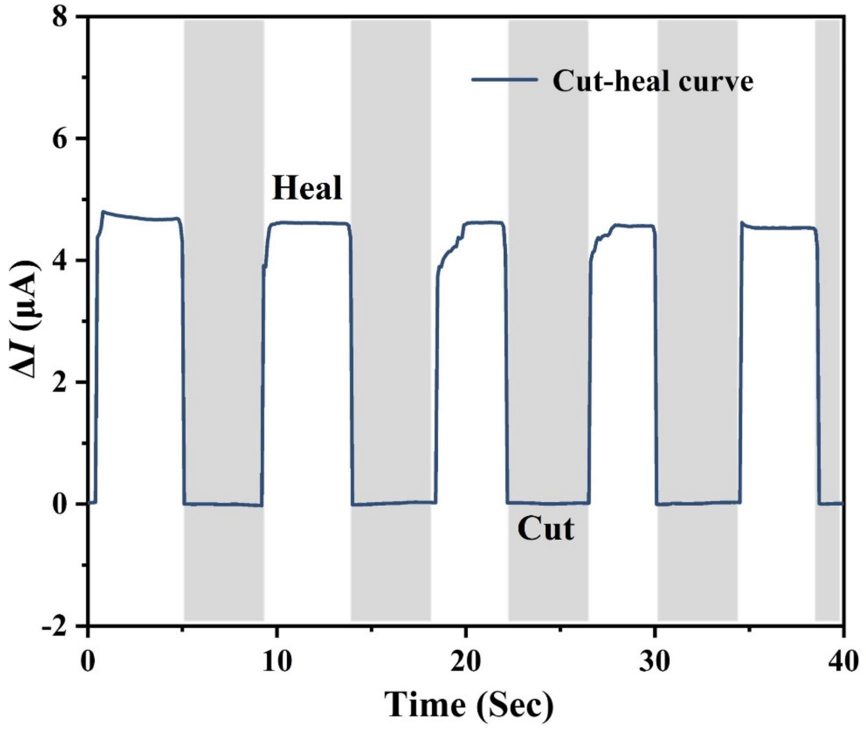

Supplementary Figure 20. Current responses in the cycle of cutting and healing.

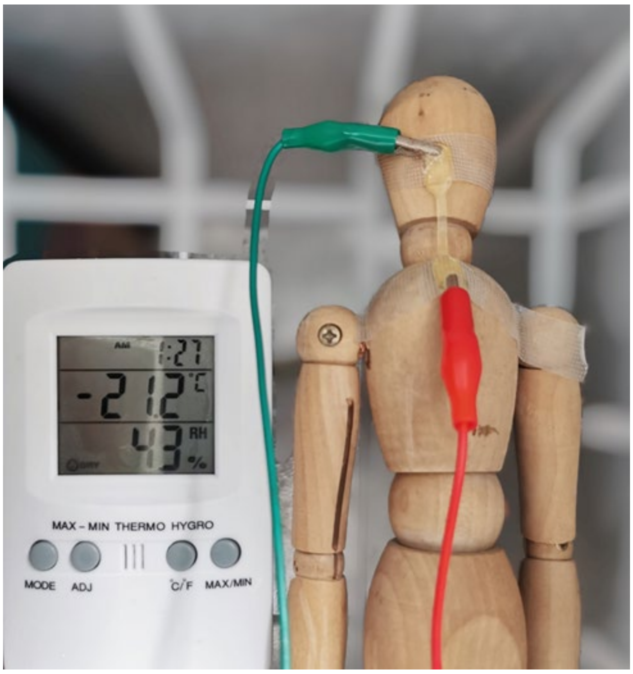

Supplementary Figure 21. The digital photograph of sensing ability test at subzero temperatures.

Supplementary Table:

Table 1. Comparison of ultimate tensile strength, self-healing abilities, and functional healing of various self-healing polymers [a].

Ref.	Self-healing motif	Ultimate tensile strength [MPa]	Minimum healing temperature	Self-healing below 0 °C	Best Self-healing efficiency [%]	Functional healing
1	Dynamic covalent urea bonding	7	70	\	90	\
2	Disulfides	5.3	60	\	43	\
3	Hydrogen bonding and electrostatic interaction	0.9	25	\	20	yes
4	Hydrogen bonding and borate ester bonding	0.2	25	\	99.6	yes
5	Metal–ligand	16	25	\	99	\

6	Hydrogen bonding	18.5	25	\	77.5	\
7	Hydrogen bonding	12.9	25	\	100	\
This work	Hydrogen bonding and metal–ligand	30.9	-20	yes	97.3	yes
[a] self-healing efficiency = $\sigma_{healing}/\sigma_{original}$.						

Supplementary References

1. Wang, Z. *et al.* Dynamic covalent urea bonds and their potential for development of self-healing polymer materials. *J. Mater. Chem. A* **7**, 15933–15943 (2019).
2. Chang, K., Jia, H. & Gu, S. Y. A transparent, highly stretchable, self-healing polyurethane based on disulfide bonds. *Eur. Polym. J.* **112**, 822–831 (2019).
3. Wu, X., Luo, R., Li, Z., Wang, J. & Yang, S. Readily self-healing polymers at subzero temperature enabled by dual cooperative crosslink strategy for smart paint. *Chem. Eng. J.* **398**, 125593 (2020).
4. Song, M. *et al.* Constructing stimuli-free self-healing, robust and ultrasensitive biocompatible hydrogel sensors with conductive cellulose nanocrystals. *Chem. Eng. J.* **398**, 125547 (2020).
5. Zhang, W., Wu, B., Sun, S. & Wu, P. Skin-like mechanoresponsive self-healing ionic elastomer from supramolecular zwitterionic network. *Nat. Commun.* **12**, (2021).
6. Wang, H. *et al.* Room-temperature autonomous self-healing glassy polymers with hyperbranched structure. *Proc. Natl. Acad. Sci. U. S. A.* **117**, (2020).
7. Xu, J. H., Chen, J. Y., Zhang, Y. N., Liu, T. & Fu, J. J. A Fast Room-Temperature Self-Healing Glassy Polyurethane. *Angew. Chemie - Int. Ed.* **60**, 7947–7955 (2021).
