# Supplementary material for: Ultrarobust subzero healable materials enabled by polyphenol nano-assemblies
Source: Nat Commun. 2023 Feb 13;14:814. doi: 10.1038/s41467-023-36461-9 (PMC9925762; doi:10.1038/s41467-023-36461-9)
Supplement: Supplementary file 2 — Description of Additional Supplementary Files [file 41467_2023_36461_MOESM2_ESM.pdf]

Description of Additional Supplementary Files

File Name: Supplementary Data 1
X-ray spectroscopy (XPS) results of the polyphenol nano-assemblies

File Name: Supplementary Data 2
Laser confocal Raman microspectroscopy results of the polyphenol nano-assemblies

File Name: Supplementary Data 3
Temperature-dependent FTIR of the dynamic multiple hydrogen-bonding networks

File Name: Supplementary Data 4
Dielectric loss data at different temperatures of PDES-PEGDA, PDES-EAN-a, PDES-EAN-b and PDES-EAN-c

File Name: Supplementary Data 5
Mechanical and self-healing properties of PDES-PEGDA, PDES-EAN-a, PDES-EAN-b and PDES-EAN-c

File Name: Supplementary Data 6
Test results of strain-sensing abilities

File Name: Supplementary Movie 1
Movie of subzero self-healing ability test for PDES-EAN-b

File Name: Supplementary Movie 2
Movie of subzero self-healing ability test for PDES-PEGDA

File Name: Supplementary Movie 3
Movie of subzero self-healing ability test for PU/CD-SG and CNCs-PDES
